# Correcting the Temperature Influence on Soil Capacitance Sensors Using Diurnal Temperature and Water Content Cycles

**DOI:** 10.3390/s120709773

**Published:** 2012-07-18

**Authors:** André Chanzy, Jean-Claude Gaudu, Olivier Marloie

**Affiliations:** INRA, UMR 1114 Environnement Méditerranéen et Modélisation des Agro-Hydrosystemes, Site Agroparc, 84914 Avignon Cédex 9, France; E-Mails: gaudu@avignon.inra.fr (J.-C.G.); marloie@avignon.inra.fr (O.M.)

**Keywords:** soil water content, capacitance probe, temperature, dielectric permittivity

## Abstract

The influence of temperature on the dielectric permittivity of soil is the result of counteracting effect that depends on the soil's composition and mineralogy. In this paper, laboratory experiments showed that for a given water content, the soil dielectric permittivity was linearly related to the temperature, with a slope (α) that varied between samples taken in the same soil. These variations are difficult to predict and therefore, a simple and straightforward algorithm was designed to estimate α based on the diurnal patterns of both the measured dielectric permittivity and the soil temperature. The underlying idea is to assume that soil water content variations can be known with a reasonable accuracy over an appropriate time window within a day. This allows determining the contribution of the soil water content to the dielectric permittivity variations and then, the difference with the observed measurements is attributed to the soil temperature. Implementation of the correction methods in a large number of experiments significantly improved the physical meaning of the temporal evolution of the soil water content as the daily cycles for probes located near the surface or the long-term variations for more deeply installed probes.

## 1. Introduction

Among the sensor types used to monitor soil water content, capacitance probes (CP) that measure the soil dielectric permittivity are increasingly being used as a proxy of the soil water content. The availability of various CP models on the commercial market, their low cost and the possibility to automatize the measurements in field conditions help explain the success of this technique. However, the soil dielectric permittivity measured by CP is not only influenced by the water content, but also by other factors, such as soil mineralogy, density, conductivity and temperature. Near the surface, the soil temperature varies significantly over time and therefore can affect the diurnal patterns of CP measurements. In deeper layers, long-term temperature variations lead to an additional trend in the measurements. It is necessary, then, to apply a correction to remove the temperature effect from the capacitance probe signal.

Temperature influences the dielectric permittivity of soil through counteracting processes. An increase in temperature increases the thermal agitation of the free water molecules, thus reducing the ability of the molecules' dipole moment to align with the applied electric field, which in turn reduces the medium polarization, leading to a decrease in the dielectric permittivity. In contrast, an increase in temperature helps to break the electric forces binding water to the soil matrix, reducing the proportion of bound water and thus increasing the dielectric permittivity. At frequencies lower than 100 MHz, the strong impact of the Maxwell Wagner (MW) polarization on soil dielectric permittivity has been shown in [1]. The MW polarization occurs when free charge displacements are limited by the presence of air/liquid, liquid/solid discontinuities leading to a charge accumulation at the different interfaces between the soil phases. As the electric conductivity of the soil solution is related to the number of free charges, it has then an impact on the dielectric permittivity [1,2]. Moreover, the soil solution electric conductivity increases with temperature [3] thanks to a better charge mobility, leading to an increase in dielectric permittivity as shown in [1].

The temperature dependence of the dielectric permittivity has been shown in numerous studies [4–16]. There is a clear consensus that the temperature has a significant effect on the soil dielectric permittivity. The magnitude of such variations is equivalent to a variation of soil water content that can be greater than 0.04 m^3^m^−3^, when a standard range of soil temperature variations (10–20 °C) is considered. It is also clear that the soil texture has an effect on the dielectric permittivity's sensitivity to temperature. With sands [10–12,14], the sensitivity is smaller and sometimes the dielectric permittivity is negatively correlated to the temperature. This is consistent with the fact that the amount of bound water is small, so the temperature effect is dominated by that of the free water. With other soils, the sensitivity increases with clay content [10,12,14], which is correlated to the amount of bound water. The influence of soil water content is not clear. In all cases, the sensitivity is low in dry conditions. When the soil becomes wetter, there can either be an increase [10] or a decrease in sensitivity [7,17] with soil having a similar texture.

The decrease in dielectric permittivity of the free water with temperature is described by the Debye formula, in which the relaxation frequency and the static dielectric permittivity are related to the temperature by empirical relationships [3,18]. The dependence of the bound water fraction to the soil temperature is much more difficult to represent. Physical models have been proposed [19–21], which quantified the bounded water fraction from the temperature and the specific soil area. From a spectrum analysis of the Time Domain Reflectometry (TDR) signal and after a calibration phase, it is shown in [22] that the parameters involved in the dielectric losses representation can be optimized and then temperature effects can be removed. However, such an approach needs a calibration phase using TDR equipment, which can be considered as a strong constraint when CP are used. Also, in the range of frequencies used by the capacitance devices, the MW effect needs also to be accounted for. A model that represents the MW effect in a soil dielectric permittivity model is proposed in [23]. It involves geometrical factors that may be difficult to handle in an operational context and the knowledge of the soil solution electric conductivity, which is an additional quantity to characterize.

To overcome the difficulties in implementing soil dielectric models, empirical relationships using temperature as a cofactor have been proposed [4,6,7,10–12,14]. These relationships are linear in most of the studies. It has been shown in [23,24] that sensors operating at low frequencies can be strongly influenced by soil conductivity and therefore the temperature. Such an effect may be very strong and variable in saline soils and then taking it into account is a critical issue. This has led manufacturers to propose electronic corrections to partly balance its effect [7], which may vary with the temperature. Therefore, it would be difficult to give a physical interpretation to an empirical correction factor that includes both instrumental features and the soil dielectric permittivity response to temperature. Moreover, [25,26] have found that heterogeneities in the probe volume of influence have an impact on soil water content field calibration. Similarly, in [27,28], it is concluded that the strong spatial variability obtained with electromagnetic sensors as CP, is the result of small scale soil structures on the measured signal. As the temperature effect is related to the actual soil component distribution in the volume of influence, it is foreseen that local heterogeneities may have an impact on soil dielectric permittivity sensitivity to the temperature that needs to be defined at the probe-soil level rather than the soil level alone.

The first objective of the paper is to show the impact of soil structure on the soil dielectric permittivity sensitivity to the temperature. This was done in well controlled laboratory conditions. The second objective is to propose and evaluate a new temperature correction method for soil dielectric permittivity sensors. According to preliminary results [29], the correction method is based on *a priori* knowledge of diurnal variations of soil water content and requires temperature measurements. The correction method uses a linear relationship between the soil dielectric permittivity and the temperature, which slope (*α*) is determined empirically using dielectric permittivity and temperature measurements only.

## 2. Material and Methods

### 2.1. Laboratory Measurements

To show the variability of the *α* factor for a given soil, we sampled six undisturbed soil cores extracted in PVC cylinders (15-cm diameter, 8-cm height) in a silty clay loam soil having a homogeneous texture (25% clay, 11% sand). Three replicates (S1, S2, S3) were extracted near the surface in the ploughed layer. The dry bulk density of the samples was 1.3 Mg/m^3^. The other three (D1, D2, D3) were collected below the ploughed layer, which was denser, with a dry bulk density of 1.6 Mg/m^3^. Each sample was brought to an intermediate water content condition (∼0.23 m^3^m^−3^ for S1, S2 and S3; ∼0.275 m^3^m^−3^ for D1, D2, D3) and to a wet condition (∼0.36 m^3^m^−3^ for S1, S2 and S3; ∼0.33 m^3^m^−3^ for D1, D2, D3). To change the soil water content level, the soil cores were wetted using a suction table and then the upper side was left opened for drying. Then the two sides were sealed to homogenize the sample water content through water redistribution. Two micro-tensiometers were installed above and below the capacitance probe to check the homogeneity of the soil water content within the soil sample.

**Figure 1.**
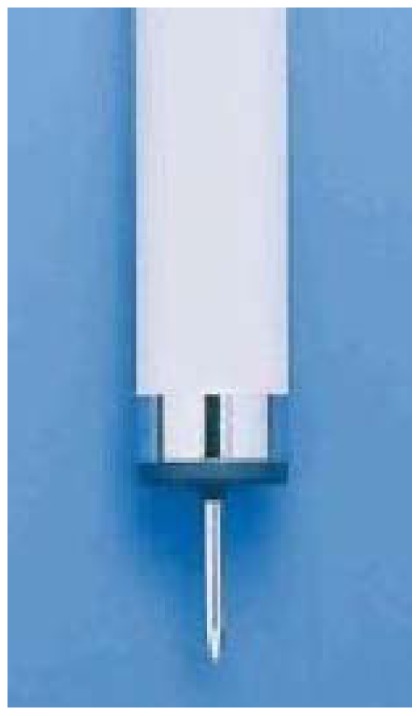
The HMS 9000 electrode geometry.

The soil dielectric permittivity measurements were performed with HMS 9000 (SDEC, Reignac sur Indre, France) capacitance probes operating at 38 MHz. They were formed by two stainless steel electrodes. Both electrodes are located along the same axis. One electrode is annular and has a length of 10 mm and a 22 mm diameter (Figure 1). The other is a rod (20 mm long, 2 mm in diameter) located 3 mm apart the annular electrode [30,31]. After being calibrated in reference media, the probe gives an apparent dielectric permittivity. The capacitance probe was installed in the middle of the sample core through a lateral access hole opened in the cylinder wall. Once the required water content was obtained, the cylinder and the sensors were installed in a thermostatic chamber to control and vary the soil temperature. The temperature was measured with a thermocouple located in the vicinity of the electrodes.

### 2.2. Principle of the Temperature Effect Correction

**Figure 2.**
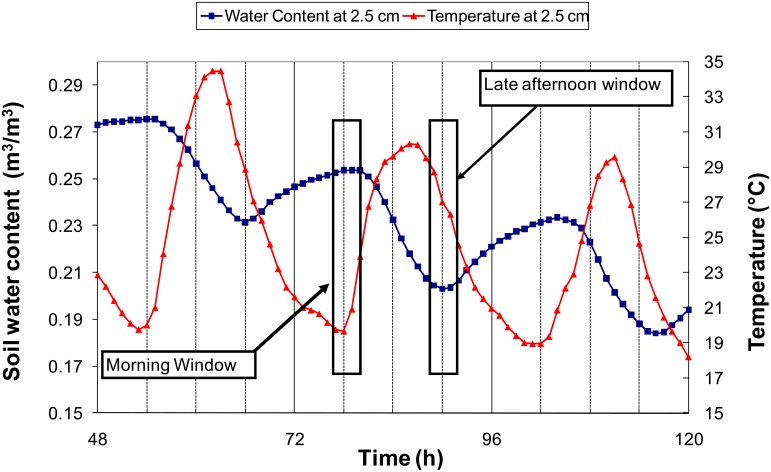
Concurrent simulated variations of soil water content and soil temperature during a drying cycle as calculated by the TEC model for the silt loam soil (CO-SiL). Solid grids at 48, 72 and 96 h correspond to midnight.

**Figure 3.**
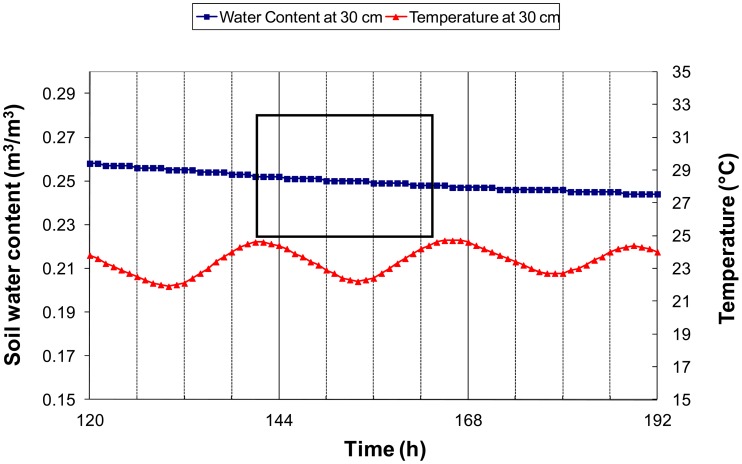
Concurrent simulated variations of soil water content and soil temperature during a drying cycle as calculated by the TEC model for the silt loam soil (CO-SiL). Solid grids at 120, 144, and 168 h correspond to midnight.

Near the surface, both the soil water content and soil temperature present diurnal cycles [32]. During the daylight, evaporation is not balanced by capillary rises leading to a decrease in soil water content. During the night, water flows near the surface are dominated by capillary rises, allowing a rewetting of the surface layer. These cycles at a depth of 2.5 cm are illustrated in Figure 2 with data simulated by the Transferts Eau et Chaleur (TEC) model, which was found to offer a good representation of the soil water content and temperature diurnal patterns [32]. There were periods of rather constant water content while the temperature rapidly changed. These periods occurred either in the early morning, just before the sunrise or in the late afternoon, when solar radiation becomes low. Below a 10-cm depth, the soil water content was only mildly influenced by the diurnal cycle. One can see in Figure 3 that at 30 cm, the soil water content decreased monotonically, while the diurnal amplitude of the temperature was about 2 °C. Moreover, it can be seen that during windows shorter than 24 h, the water content variations were roughly linear with time.

The rationale behind the temperature correction method is that during given periods, soil water content variations can be reasonably determined. Knowing such variations, one can attribute the unexpected variations of the dielectric permittivity to the temperature. Consistently with studies reported in introduction, it is assumed that the soil dielectric permittivity is linearly related to the soil temperature for a given soil water content. The aim of the method, then, is to determine the correction factor *α* to compute the dielectric permittivity at a given reference temperature (*T_r_*) using the following relationship:

(1) $${ɛ}_{Tr}={ɛ}_{m}+\alpha \cdot ({Τ}_{r}-{Τ}_{m})$$

where *ε_m_* is the dielectric permittivity measured at the actual temperature *T_m_* and *ε_Tr_* is the dielectric permittivity at *T_r_*. As reported in the introduction, *α* may vary with soil water content and the soil electric conductivity. In this study, we estimate a daily value of *α*, assuming it remains constant over a 24 h period. Four approaches were tested to estimate *α*. For all of them, we removed the day after rainfall to avoid periods of strong drainage flows. Drainage flow under wet conditions may compromise the soil water content variation hypothesis, resulting in confusion between the variations in soil water content and temperature. According to soil properties, drainage flow may last longer and therefore, removing period longer than one day after rainfall might be necessary. The methods are defined as follows:

Cm: in the early morning, the soil water content is assumed to be constant during a time window around sunrise, so *α* is estimated by the slope of the relation between *T_m_* and *ε_m_* in the considered period.
Ce: the same as Cm, but using a time window around sunset.
Cme: Both Ce and Cm are applied, and *α* are averaged. The results are then filtered by retaining days when both values of *α* are close enough (a threshold of 0.02 K^−1^ was applied to the absolute difference). More robust results using this approach are expected because the occurrence of unexpected water content variations is reduced. As a matter of fact, to fulfill the filtering criteria when soil water content varies, the soil water content variations of the morning window must balance that of the evening window.
Cd: in a given day, thanks to diurnal temperature cycles, one can find two times (*t_1_* and *t_2_*) characterized by the same soil temperature (*T*(*t_1_*)). Between these times, the soil water content is assumed to vary linearly with time as shown in Figure 3. From the probe measurements *ε_1_* and *ε_2_* performed at time *t_1_* and *t_2_*, one can determine at any time *t* between *t_1_* and *t_2_* the reference dielectric permittivity (*ε_ref_*(*t*)) at temperature *T*(*t_1_*) by the following relationship:
  (2) $${ɛ}_{\text{ref}}(t)={ɛ}_{1}+({ɛ}_{2}-{ɛ}_{1})\;(t-t_{1})/(t_{2}-t_{1})$$

The difference between *ε_ref_*(*t*) and the actual measurement *ε_m_*(*t*) results from the influence of factors other than soil water content. Among those factors, temperature during the day is likely the dominant factor. Thus, *α* is determined by computing the slope of the relationship between *ε_ref_*(*t*) − *ε_m_*(*t*) and *T*(*t*) − *T*(*t*_1_).

With all correction methods, to avoid days when the soil water content variation hypothesis is not fulfilled, we applied a filter by considering only the days when the relationship between the measured dielectric constant and temperature had a correlation coefficient higher than a threshold (here R^2^ > 0.90). With the Cme method, R^2^ is the average of that of the Cm and Ce methods. With the Cd method, *ε_ref_*(*t*) − *ε_m_*(*t*) is considered in the relationship instead of the measured dielectric constant.

It is important to note that the correction methods are only based on CP records and soil temperature measurements covering the diurnal cycle. Temperature measurements must be representative of the soil volume investigated by the CP. A temperature probe integrated with the CP is then the best measurement configuration. The main interest of the proposed correction methods is that they do not need any additional information on soil properties. The only parameters of the methods are related to the determination of the morning and evening time windows and the filtering criteria, *i.e.*, the R^2^ threshold and the number of days to remove after rainfall. For the morning and evening time windows, optimal values were determined empirically by minimizing the *α* variability and maximizing the R^2^ between the CP signal and the temperature. It led to select a two hour time windows starting one hour after sunrise and two hours before sunset for the Cm and Ce methods, respectively. The time windows are implicitly defined as periods, during which water flows are conservative within the soil layer characterized by the CP. Such periods may change with the depth of the sensor and the soil properties. Adjustment of the time window could be then necessary.

### 2.3. Simulations

All correction methods (Cm, Ce, Cme and Cd) are based on hypotheses made on the soil water content variations. However some deviations from the expected soil water content variations may have an impact on the determination of *α*. To evaluate this impact, records of soil water content and temperature that covered a range of diurnal patterns are necessary. These patterns are influenced by soil hydraulic properties, soil depth and climate conditions. Moreover to evaluate the error in *α*, its “true” value must be known. To obtain such records, we used soil water content and temperature data simulated by the TEC model [32], which represents the vertical soil water and heat flow according to Philip and de Vries coupled equations [33]. The boundary conditions of such equations can be determined using standard climatic observations and heat and water flows in liquid and gaseous phases are considered, which are the main fluxes near the surface. Four soils covering a wide range of soil textures were taken from the data base described in [34]: Co-SiL [silt loam with sand fraction (S) S = 38.8%; Clay fraction (C) C = 10.5% ], Al-SiL (silt loam with S = 34.4, C = 17.0%), Po-SiCL (Silty Clay Loam with S = 11.0%, C = 27.2%) and Al-SiCL (Silty Clay loam S=5.3%, C = 38.9%]. Al, Po, and Co corresponds to soil origin in agreement with soil description made in [34]. All the simulations were made over a 90-day period, which was characterized by contrasting weather conditions, with heavy rains and long periods of drying.

Within the time windows used to determine *α*, the impact of unexpected soil water content variations depends on the difference between the expected water content and its true value (here the simulated water content) and the sensitivity of *ε* to the soil water content (*θ*). The resulting error in *α* increases when both the difference between expected and actual *θ* and the sensitivity of *ε* to *θ* increase. To maximize the impact of soil water content hypothesis failure on *α* determination, we considered an *ε* to *θ* sensitivity of 80 to transform simulated *θ* into *ε*. It corresponds to the sensitivity of the free water, while free/bound water partition generally reduces the sensitivity of *ε* to *θ*. Such maximization is effective for most soils, with the exception of clayey soils at low frequencies that may present higher sensitivity of *ε* to *θ* [24].

The instrumental noise also alters the capacity of detecting temperature and CP signal variations. To represent it, an unbiased Gaussian error was added to *ε* and *T*. The standard deviations were set to 0.001 and 0.05 for *ε* and 0.01 K and 0.05 K for *T*. These values were selected according to the observed noise on temperature and CP measured signals that can be filtered by averaging data. The standard deviations can be found optimistic if absolute accuracy of the sensors is considered, but here, the correction methods are based on signal variations only.

### 2.4. Field Measurements

We gathered data from different field experiments [34–36]. Measurements were made by the HUMICAP (SDEC, Reignac sur Indre, France) probes before 1997 and then with its upgraded version, the HMS9000. Most of the measurements were taken on tilled fields within the surface soil layer (0–20 cm) with the probes installed horizontally. Some measurements were taken in deeper layers down to 100 cm with a vertical installation. Temperature measurements were made with platinum resistance thermometers (resolution: 0.01° K, accuracy 0.5° K) installed in the capacitance probe's vicinity and at the same depth, when HUMICAP sensors were used. The HMS9000 probe integrates a temperature probe, which was used for the temperature corrections. Correction factors were computed for every sequence of measurements made after the probe installation. This means that a new correction factor was determined when a probe was removed and reinstalled. The results from the 100 sequences reported in this paper were acquired from different soils with a clay content ranging from 13.6% to 48%.

To assess the variability of the temperature coefficient on a given site and to use another type of CP sensor to demonstrate that the correction method can be applied to a range of sensors, we used data from the INRA-Avignon observatory (France), soil water content, temperature and surface energy flux data have been collected continuously over a long period (measurements made since 1998) on a cultivated field. The soil is a calcaric luvisol with a silty clay loam texture (33% clay, 54% loam and 13% sand). In this experiment, three EC-10 (DECAGON, Pullman, WA, USA) probes, operating at 5 MHz, were installed horizontally within the 0–5-cm layer. These probes were removed before every tillage operation, and they were reinstalled afterwards. The probes were powered by a 2.5-V excitation. To convert the measured signal (*M*) into a dielectric permittivity, the probes were calibrated using different liquids that had a known dielectric permittivity (from 6 to 47). The following empirical relationship was obtained:

(3) $$ɛ=0.495\cdot e^{0.00627\cdot M-0.221}$$

The RMSE of the retrieved *ε* was 1.4. Temperature was measured at the depth of the probe using a platinum resistance thermometer installed near the CP. Eight sequences of measurements collected during the 2003–2008 period were considered, with period lengths ranging from 105 to 262 days.

## 3. Results and Discussion

### 3.1. Temperature Influence on Capacitance Probe Measurements

The results of the laboratory measurements of the temperature influence on the soil dielectric permittivity are displayed in Table 1. A linear regression was used to estimate the sensitivity (*α*) of the dielectric permittivity to the temperature for all samples and for every water content level (results are given in Table 1). In most cases, the R^2^ was greater than 0.9, which means that a linear model provided a suitable representation of the dielectric permittivity's dependence on temperature. The R^2^ was however smaller with the wet D2 and S1 samples. With the S1 sample, a α value close to zero explains the low R^2^, whereas for the D2 wet samples, hysteresis appeared during the experiment between the warming and cooling phases. Equilibrium may not be always obtained with the wet samples.

The slope for a given soil material was rather variable from one sample to another (from 0.006 K^−1^ to 0.14 K^−1^). This variability may be explained by the variable soil structure in the probe's sphere of influence, which only covers a volume of a few cm^3^ [30] and variations in soil water content and electrical conductivity. At this scale, the soil structure in the vicinity of the electrodes can change drastically from one probe to another. Therefore, the proportions of bound and free water detected by the probe can vary, and consequently, the dependence of the dielectric permittivity on the temperature may also reflect this variability.

The results displayed in this section show a clear effect of the temperature on the soil dielectric permittivity, which needs to be accounted for when deriving soil water content from capacitance probe measurements. The obtained *α* variability prevents the use of a single representative value for a given soil, and a probe by probe determination of *α* must be done. As the soil moisture also impacted *α*, as shown with the lower values obtained in wet conditions, this determination should be done at the different moisture levels.

### 3.2. Correction Method Evaluation on Simulated Data

**Figure 4.**
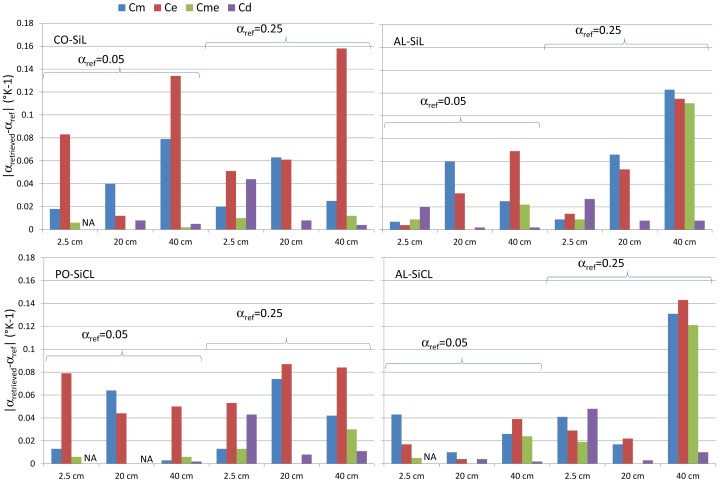
Absolute difference between the retrieved α (K^−1^) at several depths (2.5, 20 and 40 cm) and the reference α (α_ref_) determined from the data simulated by the TEC model using the four methods (Cm, Ce, Cme, Cd). Each graph corresponds to a given soil. NA is given when *α* could not be determined. SiL and SiCL correspond to soils having a Silt Loam and Silty Clay Loam texture, respectively.

In the evaluation of the correction method, we considered two levels of *α*, 0.05 and 0.25 °C^−1^, which bracketed experimental results as displayed in Table 1. No errors were added to *T* and *ε* in the first step. The estimation methods were applied to the whole 90-day simulation, and the average *α* coefficient was computed with the data that fulfilled the filtering criteria as defined in the previous section. The four soils considered (Co-SiL, AL-SiL, Po-SiCL, AL-SiCL) only differed by their soil water content and temperature dynamics resulting from their specific hydraulic and thermal properties. The impact of the soil texture on the soil dielectric permittivity was not taken into account because a single relationship was used to compute *ε*. The results are displayed in Figure 4. It is apparent from the figure that the Cm and Ce methods were not accurate, though slightly better results were obtained with the Cm method. Much better results were obtained with the other two methods. At the 2.5-cm depth, Cme was clearly the best, whereas Cd was better at a 40-cm depth. At the intermediate depth, both Cme and Cd methods offered similar accuracy. The results appeared to be better when *α* was small. In fact, when *α* was larger, the temperature effect on *ε* was stronger, leading to a larger R^2^ (the correlation coefficient of the relationship between *ε* and T, as explained in the material and method section), because the *ε* dynamic was larger. Therefore, the loss in accuracy can be explained by the more permissive filtering, which was based on the R^2^ threshold of 0.9, below which the data were not considered. No clear effect can be observed with the different soils.

**Figure 5.**
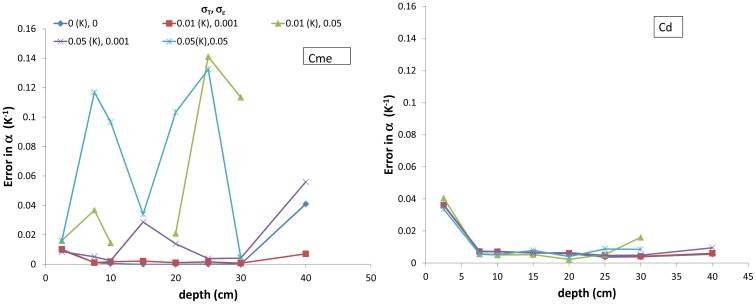
Error in α as a function of depth considering the Cme and Cd methods. Different measurement error scenarios given in the legend for temperature (σ_T_) and the dielectric permittivity (σ*_ε_*) are considered.

To assess the impact of experimental noise on the results, different levels of measurement errors were considered. To determine the error in the estimation of *α*, we proceeded as follows. As done previously, the average *α* over the 90-day simulation period was determined for every combination of soil/depth/*α*. Then, the absolute difference between the retrieved and the true *α* was computed. Finally, for a given depth and error scenario, the error was determined as the average of the absolute differences. The results are displayed in Figure 5. When the measurement errors were small (σ_T_ ≤ 0.01 °K, σ*_ε_* ≤ 0.001), Cme offered excellent results from the surface to a depth of 30 cm. Below 30 cm, the Cd method was preferable. The Cd method was also better with larger errors, except near the surface (within the top 5 cm), thanks to the larger number of measurements involved in the Cd method, which reduce the impact of the measurement errors. Increasing the sampling in time (here, hourly measurements were considered) could be a way of applying Cme with noisy sensors.

### 3.3. Retrieved Temperature Correction Factor and Impact on Soil Water Content Measurements

In this section, the Cme method was applied to the top 10 cm, and Cd was applied below that depth on different experimental data sets. Each probe was calibrated using gravimetric measurements. The independent water content measurements were made in the same field, and the calibration procedure was performed according to [36].

**Figure 6.**
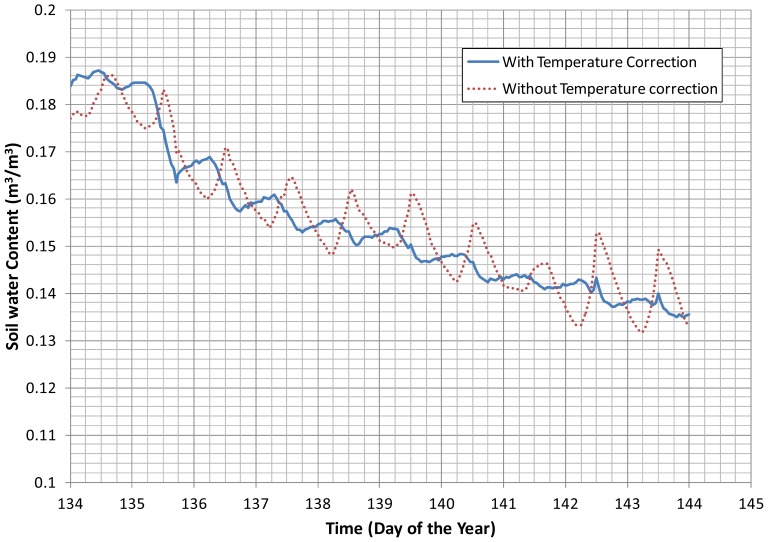
Soil water content measured by the HMS9000 located at a 2.5-cm depth. The probe calibration was done either with the unprocessed measurement (dotted line) or with the measurement corrected by the temperature effect (solid line). Solid grids at 134, 135 … correspond to midnight.

The results obtained during the Alpilles-Reseda experiment [35] with a HMS9000 probe located at 2.5 cm are displayed in Figure 6. When the measured signal was not corrected for temperature effects, the temporal patterns of the retrieved water content were not relevant. As shown previously in [15], the soil water content increased during daylight and decreased during the night, which is unrealistic. After the temperature correction (using the Cme method), the drying and wetting periods were properly phased with the climatic demand. In this case, the correction coefficient *α* was found constant and equal to 0.115 K^−1^, which is consistent with the slopes given in Table 1. One can notice the spikes in Figure 6 with the second day and the last two days at noon showing the limit of having a constant value of *α* for all soil wetness conditions. It was however difficult to identify a clear trend between α and the soil water content.

**Figure 7.**
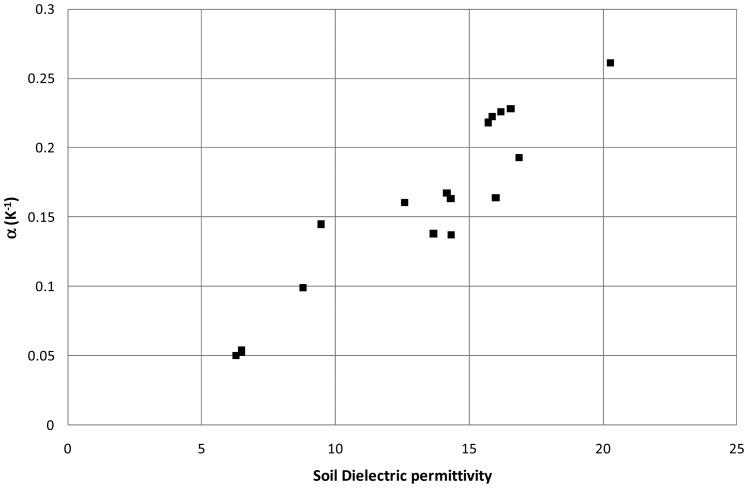
Temperature Sensitivity coefficient α as a function of the soil dielectric permittivity obtained with the DECAGON EC-10 probe installed at 2.5 cm depth at the INRA-Avignon observatory. Measurements were taken during the bare soil period of the sequence 2 with the sensor P3 (Table 2).

**Figure 8.**
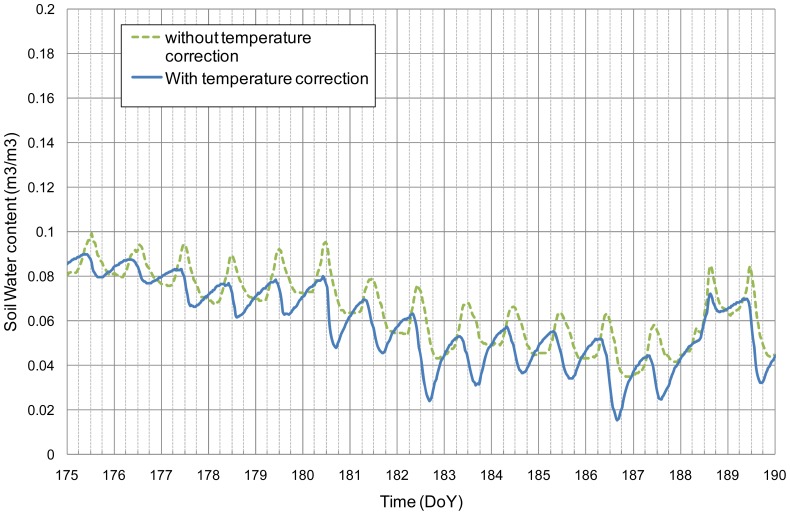
Soil water content measured by an EC-10 installed at a 2.5-cm depth. The probe calibration was done either with the unprocessed measurement (dotted line) or with the measurement corrected by the temperature effect (solid line). Vertical solid grids correspond at midnight.

The Cme method was also applied to the EC-10 measurements made on the INRA observatory site. Results displayed in Figure 7 show a strong dependence of *α* on *ε*. Such a dependence was already shown in previous studies [17,37–39]. In these studies different shapes of the *α vs. ε* relationships were found (linear, parabolic, and hyperbolic). The proposed method allows building those relationships without having any a priori analytical form. In our case, a linear relationship was fitted and applied to estimate α used to correct the temperature effects. Results of a 15 day sequence are displayed in Figure 8. The diurnal patterns of the soil water content estimated from the corrected signal are more consistent with the expected drying during the daylight and wetting during the night. The amplitude in soil water content is the greatest during day of the year (DoY) 180, 182 and 186. The more intensive drying on these days may be explained by the climatic conditions characterized by high global radiations and a small wind speed that are favorable conditions to water vapor flows in the soil and its drying within the top first centimeters [32].

**Figure 9.**
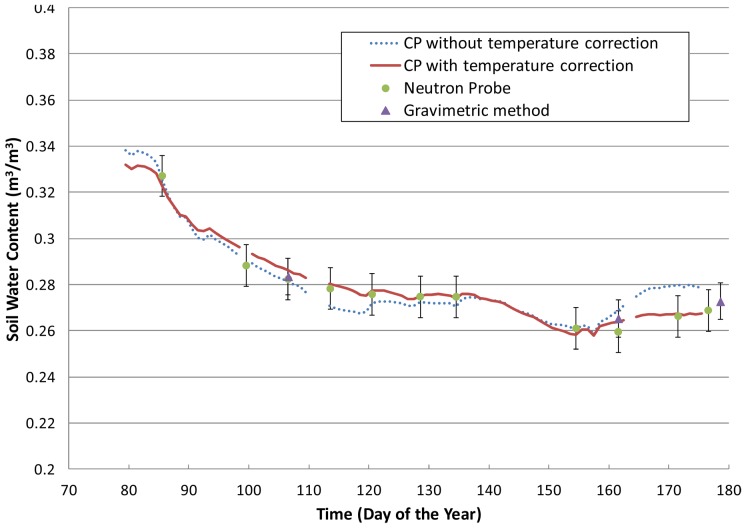
Soil water content measured by a HMS 9000 capacitance probe located at a 65-cm depth and by Neutron and gravimetric methods. Temperature coefficient *α* was determined by the Cd method. Error bars corresponds to the standard deviation of the measurements.

In Figure 9, one can see the results obtained during the Alpilles-Reseda experiment [35] with a HMS9000 probe at a depth of 65 cm. As the daily temperature amplitude is very small at that depth, the results obtained at midday over a period of 99 days are represented. Soil water contents derived from the CP measurements were compared to gravimetric measurements (average of 5 replications) and neutron probe measurements (average of 2 replications) made within the same field. Figure 9 clearly shows the impact of correcting for the temperature effect on soil water content values, especially at the end of the period when the temperature started to increase at 65 cm. The obtained value for *α* is 0.22 K^−1^, which is greater than the result displayed in Table 1. This result is consistent with the texture of the considered soil, which has a larger clay fraction (42%) than that of the silty clay loam soil used for the laboratory experiment (27%).

**Figure 10.**
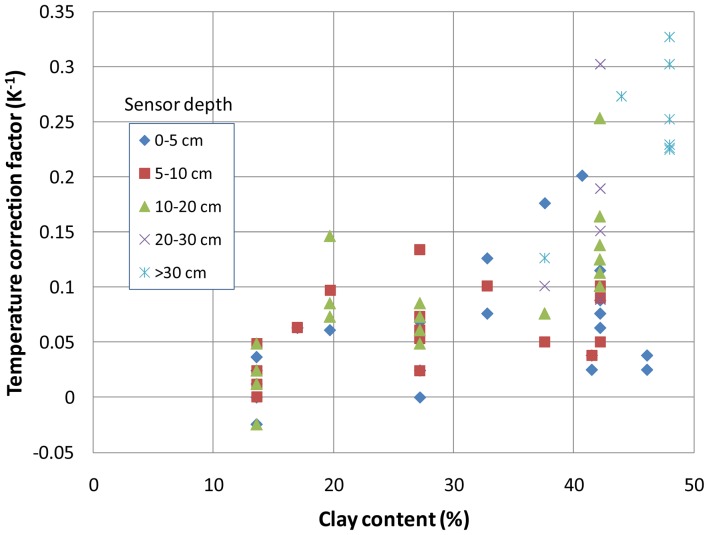
Correction factors obtained for different probe installations within different soils characterized by their clay content.

In Figure 10, the *α* obtained for 100 sequences of measurements with HUMICAP and HMS9000 sensors are displayed. The soils are identified by the clay fraction in the x-axis, whereas the depth of installation is given by the symbols. The figure shows an overall expected trend with an increase in *α* in the clay content. However, a strong variability was found for a given soil, which may result from the variability of the soil composition within a probe's volume of influence.

The results obtained with the DECAGON EC-10 at the Avignon observatory are displayed in Table 2. The coefficients of *α* were determined with the Cme method. The average *α* for a given set of sequences and a given probe ranged from 0.051 to 0.33 K^−1^. Such results are consistent with the other results presented in this study, even if they are greater than that obtained with the HUMICAP or HMS9000 probes. This result may be explained by the EC-10 probe's geometry, which allows a better soil electrode contact, or by the EC-10 operating frequency, which is lower (5 MHz). The variability of the results remains strong, as shown by the range of the average *α* values and by the standard deviation of *α* for a given measurement sequence, the ratio average/standard deviation often being higher than 50%, Such high ratios are mostly explained by the strong dependence of *α* to *ε* illustrated in Figure 7 and by the correlation coefficient given in Table 2. For sequences 6 and 7, the filtering criteria led to a strong reduction in the number of days to characterize *α*. Sequence 6 occurred during the winter period. The number of days was reduced after removing the days with a soil temperature below 0 °C (to avoid the frost effect on the dielectric permittivity). Moreover, the temperature dynamic was smaller, which may have affected the filtering based on the correlation coefficient. Sequence 7 corresponded to a period with a sorghum crop. At the beginning, frequent irrigation led to a decrease in days of acquisition. Afterwards, the *α* difference between the morning and evening periods was always slightly greater than 0.02 K^−1^. The daily patterns of the shade cast by the canopy can vary locally, so differences in soil temperature may occur between the soil temperature and the CP measurements sites, which encourages using a CP that integrates a temperature sensor.

## 4. Conclusions

We note in this paper that with the three different capacitance probe types tested, it is difficult to make a temperature correction using the physical characteristics of the soil. For a given soil, wide range of *α* was found under laboratory and field conditions. This variability may be explained by the soil structure in the probe's sphere of influence, which can be highly variable from one probe to another due to natural variability, the quality of the probe installation and the electrode size and configuration. If the design of the experiment allows for the measurement of the diurnal variations of both the soil dielectric permittivity and the temperature, we propose an efficient method to correct the temperature effect on the soil dielectric permittivity measurement. This method needs to be applied to every probe independently, and it does not require any information on soil properties. The method has been tested on a large number of cases (>100). A strong variability with the retrieved *α* values was obtained, even with probes installed in the same soil, thus encouraging the determination of *α* at the probe level after every probe's installation. The simplicity and robustness of the proposed method provides a straightforward approach to achieving this processing step.

With probes operating at 38 MHz, a single correction factor *α* was suitable to perform the correction for measurements taken over long periods of time (several months). In contrast, at 5 MHz, the dependence of *α* on the soil water content was clearly demonstrated. Because the proposed method can be implemented continuously without additional effort, it is not a problem to collect enough values to determine the relationship relating *α* to *ε* or to the soil water content conditions or to account for variations in *α* due to soil electric conductivity.

The proposed temperature correction method is generic (the R function will be made available by the authors on request). Although it was implemented with only three types of sensors, the observed impacts of the temperature on the dielectric permittivity were consistent with those obtained in previous studies based on other types of sensors. Moreover, the method is based on the diurnal variation of both the dielectric permittivity (or a proxy such as the delivered electric signal by the CP) and the temperature. These observations can be obtained with standard temperature probes and all kinds of soil water content sensors based on the characterization of the soil dielectric permittivity (CP, TDR, microwave remote sensing), provided frequent measurements throughout the day are acquired and the assumptions of the methods satisfied.

## Figures and Tables

**Table 1.** Results of the regression between the dielectric permittivity and temperature for all soil samples.

**Sample**	**Dry**			**Wet**		

	**α (K**^−^**^1^)**	**α error (K**^−^**^1^)**	**R^2^**	**α (K**^−^**^1^)**	**α error (K**^−^**^1^)**	**R^2^**
D1	0.093	0.004	0.98	0.060	0.004	0.97
D2	0.095	0.002	0.99	0.090	0.025	0.63
D3	0.140	0.005	0.98	0.127	0.004	0.99

S1	0.090	0.002	0.996	0.006	0.006	0.01
S2	0.114	0.001	0.999	0.037	0.004	0.92
S3	0.072	0.005	0.96	0.104	0.013	0.90

**Table 2.** Temperature correction factor results obtained with the three EC-10 CPs (P1, P2 and P3) and the eight measurement periods at the INRA-Avignon Observatory. Three types of data are given for *α*: the average value over the measurement period, the standard deviation (in brackets) and the number of days allowing the computation of *α*. R^2^ is the coefficient of determination between *α* and *ε*.

***Sequence Number***	***Measurement Period (d)***	***Crop***	***α̣(K****^−1^****) P1***	***α̣ (K****^−1^****) P2***	***α (K****^−1^****) P3***	***R****^2^****(α̃ε) P1***	***R****^2^****(α̃ε) P2***	***R****^2^****(α̃ε) P3***
1	105	Sunflower	0.051 (0.097) *39*	0.081 (0.023) *30*	0.064 (0.033) *31*	0.74	0.27	0.87
2	239	Winter Wheat/Bare soil	0.081 (0.041) *81*	0.217 (0.019) *16*	0.141 (0.072) *20*	0.88	0.62	0.81
3	150	Pea	0.128 (0.061) *49*	0.167 (0.132) *19*	0.19 (0.059) *12*	0.87	0.98	0.73
4	262	Winter Wheat	0.083 (0.049) *48*	0.109 (0.044) *54*	0.118 (0.042) *37*	0.78	0.72	0.66
5	111	Bare soil	0.138 (0.062) *10*	0.059 (0.039) *33*	0.152 (0.101) *22*	0.92	0.97	0.80
6	111	Bare soil	NA (NA) *0*	0.13 (0.042) *14*	0.065 (NA) *2*	NA	0.78	NA
7	152	Sorghum	0.284 (0.137) *3*	0.330 (0.063) *3*	0.200 (0.037) *8*	NA	NA	0.00
8	118	Winter Wheat	0.089 (0.041) *39*	0.133 (0.120) *23*	0.117 (0.039) *29*	0.79	0.95	0.55
